# Supplementary material for: The Role of Tailored Public Health Messaging to Young Adults during COVID-19: “There’s a lot of ambiguity around what it means to be safe”
Source: PLoS One. 2021 Oct 1;16(10):e0258121. doi: 10.1371/journal.pone.0258121 (PMC8486094; doi:10.1371/journal.pone.0258121)
Supplement: S1 Appendix — (DOCX) [file pone.0258121.s001.docx]

**Appendix A**

**Questions**:

1. What do you know about the coronavirus?
   1. Who gets it?
   2. What are the health consequences?
   3. What measures can you take to protect yourself?
   4. What do you think are the riskiest behaviours in transmitting the virus?
2. How has the pandemic affected your lives?
3. When you think of the coronavirus, what do you find the most concerning?
4. What is the most important to you with respect to returning to normal life
5. What should the government’s priorities be to deal with the pandemic?
6. How do you think other people in your age group are reacting to the pandemic?
7. Have you seen or heard media report about people in your age group violating the rules about COVID-19?
   1. What do you remember from those reports? How did they make you feel?
   2. Why do you think people in your age group might behave this way?
   3. When you hear coronavirus cases are increasing in your age group, what does it mean to you?
8. Tell me about a time that you had to make a decision that involved weighing the risks of COVID. What did you decide? How did you come to that decision?
9. How do you get your information about the coronavirus?
10. How do you distinguish between real and false information?
11. What media are best for reaching you and your peers?
12. How responsible do you personally feel to keep yourself and others healthy during the pandemic?
