# Supplementary material for: The Role of Tailored Public Health Messaging to Young Adults during COVID-19: “There’s a lot of ambiguity around what it means to be safe”
Source: PLoS One. 2021 Oct 1;16(10):e0258121. doi: 10.1371/journal.pone.0258121 (PMC8486094; doi:10.1371/journal.pone.0258121)
Supplement: Attachment — Submitted filename: COVID-YA PLOS ONE Revision Response- FINAL revised.docx [file pone.0258121.s002.docx]

Response to Reviewers

*PONE-D-21-17314
The Role of Tailored Public Health Messaging to Young Adults during COVID-19: “There’s a lot of ambiguity around what it means to be safe”
PLOS ONE*

***Journal Requirements:***

*When submitting your revision, we need you to address these additional requirements.*

*1. Please ensure that your manuscript meets PLOS ONE's style requirements, including those for file naming. The PLOS ONE style templates can be found at* 
[*https://journals.plos.org/plosone/s/file?id=wjVg/PLOSOne_formatting_sample_main_body.pdf*](https://journals.plos.org/plosone/s/file?id=wjVg/PLOSOne_formatting_sample_main_body.pdf)

*and* 
[*https://journals.plos.org/plosone/s/file?id=ba62/PLOSOne_formatting_sample_title_authors_affiliations.pdf*](https://journals.plos.org/plosone/s/file?id=ba62/PLOSOne_formatting_sample_title_authors_affiliations.pdf)

This has been addressed.

*2. Thank you for stating the following financial disclosure:*

*"The funders had no role in study design, data collection and analysis, decision to publish, or preparation of the manuscript."*

*At this time, please address the following queries:*

*a) Please clarify the sources of funding (financial or material support) for your study. List the grants or organizations that supported your study, including funding received from your institution.*

The authors received no specific funding for this work.

*b) State what role the funders took in the study. If the funders had no role in your study, please state: “The funders had no role in study design, data collection and analysis, decision to publish, or preparation of the manuscript.”*

Not applicable as no funding was received.

*c) If any authors received a salary from any of your funders, please state which authors and which funders.*

This does not apply.

*d) If you did not receive any funding for this study, please state: “The authors received no specific funding for this work.”*

*Please include your amended statements within your cover letter; we will change the online submission form on your behalf.*

The authors received no specific funding for this work.

*3. Thank you for stating the following in the Acknowledgments Section of your manuscript:*

*"We thank the participants for their generosity with their time.* *SAL holds the Pfizer/Heart and Stroke Foundation Chair in Cardiovascular Prevention Research at St. Paul’s Hospital."*

*We note that you have provided funding information that is not currently declared in your Funding Statement. However, funding information should not appear in the Acknowledgments section or other areas of your manuscript. We will only publish funding information present in the Funding Statement section of the online submission form. 
Please remove any funding-related text from the manuscript and let us know how you would like to update your Funding Statement. Currently, your Funding Statement reads as follows:*

*The funders had no role in study design, data collection and analysis, decision to publish, or preparation of the manuscript.*

*Please include your amended statements within your cover letter; we will change the online submission form on your behalf.*

This does not apply. “SAL holds the Pfizer/Heart and Stroke Foundation Chair in Cardiovascular Prevention Research at St. Paul’s Hospital.” is not an indication for funding source.

*Additional Editor Comments (if provided):
Please review your reference list to ensure that it is complete and correct. If you have cited papers that have been retracted, please include the rationale for doing so in the manuscript text, or remove these references and replace them with relevant current references. Any changes to the reference list should be mentioned in the rebuttal letter that accompanies your revised manuscript. If you need to cite a retracted article, indicate the article’s retracted status in the References list and also include a citation and full reference for the retraction notice.*

This does not apply.

***Reviewer #1****: The authors have put together an interesting and relevant report especially with regard to matters affecting young adults during the COVID-19 pandemic. The manuscript is sound. Although it is usually expected that focus groups should comprise 6-8 participants, the the 4-6 participants in this study is still acceptable. Perhaps, the authors should replace the word 'stakeholds' with ' stakeholders' such as in the conclusion. Overall, this research is good, and should be published*

Thank you for these positive comments. The word ‘stakeholds’ had been replaced with ‘stakeholders’ in the conclusion [page 21].

***Reviewer #2****: This is a timely and very-well conducted and reported focus group study. In particular, the description of focus group methodology and coding process are exemplary. I have a couple of larger points and some minor stylistic points that should be addressed to further improve the manuscript, but have little doubt that the authors will be able to do so.*

Thank you for these positive comments.

*Major points:
1. I would like to see more of a justification for the use of focus groups. The manuscript nods to some drawbacks (like stronger social-desirability bias) and make use of some of the more interactive characteristics of the method, but I’d like to see more of a weighing of different options of collecting qualitative data and their suitability for the questions of interest (e.g., why were focus groups deemed a better model than semi-structured interviews?)*

In comparison to other qualitative data collection strategies, focus group discussion has the strength in providing access to the shared social meanings or norms and how they are performed. As the objective of this study was to understand the perceptions of young adults on the existing public health messaging and their lived experiences, the focus group method compared to other qualitative data collection strategy, is more able to resemble the natural interactions participants might have in their everyday lives. While individual interviews may have higher potential for insights, we believe it is essential to explore how interaction dynamics may influence young adults’ perceptions about current messaging. Focus group discussion allows us to explore how young adults communicate about the current messaging and examine what tailored messaging to this demographic would look like.

Highlighting why focus group discussion was chosen in comparison to other qualitative methods, this sentenced was added to the Method section on page 5.

“The focus group discussion methodology was chosen for its strength in providing access to in-depth understanding of shared social meanings or norms. Through focus group discussions, we aimed to explore the natural interactions participants might have in their daily lives and how these interactions may influence their perception of existing health messaging.”

*2. Especially in the discussion, the manuscript should be more circumspect about the generalizability of their findings – which is minimal. As noted under limitations, the sample is not representative and, given recruitment strategy, likely significantly biased towards those more informed (and likely more concerned) about COVID-19. For example, the study methodology simply doesn’t permit statements such as “Overall, we found young adults, contrary to mass media coverage, were highly concerned about the spread of COVID-19and perceived it as their responsibility to protect themselves and their loved ones.” (p. 14 – there are a number of other similar examples but this is highlighted as one of the study’s principal findings). Instead, I would encourage the authors to focus on the themes around messaging and perceptions (as a good bit of the discussion already does), where qualitative insights are of particular richness and value.*

Overall, the young adults in our study were highly concerned about the spread of COVID-19 in contrast to their portrayal in mass media coverage. They also perceived it as their responsibility to protect themselves and their loved ones.

Our discussion's goal was to highlight the three main features that emerged from thematic analysis that seemed unique to our participants in this demographic that future public health messaging should consider when designing tailored young adults messaging. We have re-read the Discussion and tempered some of the concerning statements as directed. For example:

Page 15: Overall, our participants, contrary to mass media converge, were highly concerned about the spread of COVID-19 and perceived it as their responsibility to protect themselves and their loved ones.

Page 17: Almost all young adults who participated in focus group discussions were aware of media coverage on events caused by people in their age group that resulted in community spread.

*3. The authors’ use of “thematic analysis” does not correspond to Braun and Carke’s version of this (see their 2019 article [1], including the note that notions of intercoder reliability and multiple coders are irreconcilable with “reflexive thematic coding.” As the 2019 update helpfully clarifies, there are other, more positivist approaches to TA that are more appropriately referenced here.*

Thank you for directing us to the 2019 Braun & Clarke paper. Indeed, intercoder reliability would not be consistent with "reflexive thematic coding," as clarified by the authors. There are other, more positivist approaches we can cite, such as Boyatzis (1998) or Joffe (2011), if we intend to show a measure of the consistency of judgment across two coders.

1. Boyatzis, R. E. (1998). Transforming qualitative information: Thematic analysis and code development. Thousand Oaks, CA: Sage.

2. Joffe, H. (2011). Thematic analysis. In D. Harper & A. R. Thompson (Eds.), Qualitative methods in mental health and psychotherapy: A guide for students and practitioners (pp. 209-223). Chichester: Wiley.

After careful consideration and reflecting on how themes were developed as a group, both qualitative coders agreed the process of finding codes and the development of themes were essentially inductive, with the process being flexible and organic. Themes were generated as patterns of shared meaning on an abstract level and not domain summaries of what participants said during the focus group. The themes in this study were analytic outputs created from codes and through the researcher's active engagement with the data and ongoing discussions between team members. Cohen's Kappa was calculated initially to discuss and discover assumptions or if coder overlooked the data individually. Yet, coding was largely based on ongoing discussions between the two coders. Ways we used to ensure reliability include paying close attention to rigor in fieldwork, including accurate notetaking, transcribing verbatim, and discussing emerging coding with colleagues throughout. Codes were used as building blocks for the development of themes rather than evidence for previously developed themes. As both coders believe there is no one "accurate" way to code data, the team believes the kappa score should be removed to reflect the honest process of how themes were developed. The interrater reliability has been removed from the manuscript.

*Minor points:
1. It would be helpful to very briefly outline the COVID-19 situation and government response in Canada at the time of the FGs. That’s most relevant with respect to economic pressures faced by participants, but also to better understand their assessment of the government’s response and communication strategy*

More context information had been added in Discussion to give more information on the government response during the time of the FGs.

Page 17

During the time of the focus group, the province had been in a state of emergency for approximately eight months, with changing public health orders restricting social gatherings, traveling, and indoor activities. The province had been in full lockdown since early spring of 2020. While the provincial restrictions had relaxed for the summer of 2020 following the province’s restart plan, the declaration of a second wave in October amended further strict regulations on gatherings. Furthermore, the provincial health officer issued two-week regional lockdown measures, including restricting all social gatherings of any size. While public services such as schools remained open, local small businesses such as restaurants and dance studios suspended operations according to changing health orders. A provincial mask mandate was issued shortly after the focus groups were conducted.

*2. Figure 1 is a bit odd – I understand these are the four main themes, but does the way they’re arranged and connected have any meaning? Based on the text, I don’t think so. If they don’t, maybe just putting this in a table (perhaps even with a bit more definition) might be clearer*

Figure 1 had been switched to Table 3, hopefully this presents the summary of themes in a clearer fashion. This table can be found on page 7- 8.

I: Risk of Disease Contraction: participant’s perception of actions that would increase their risk of contracting the virus

II: Impact of COVID 19

- Epidemiology: participant’s perceptions on features and information of the virus
- Individual: both biological and psychological impact the pandemic has on an individual
- Societal: perceived short- and long- term impact of the virus on society, with a focus on economic pressure faced by local small businesses

III: Role of institutions

- Media: participant perception on the function and purpose of media during the pandemic
- Government: participant perception on the responsibility and action of the government during the pandemic
- Individual: responsibility an individual should take to lower transmission during the pandemic

IV: Effective Public Health Messaging

- Information Seeking and Receiving: where young adults actively seek public health information and where they receive such information passively
- Suggestions for Effective Messaging: concerns and questions participants had regarding current messaging lead to suggestions for future messaging

*3. The percentage figures in table 1 are confusing. For the first column, they add up to 100% vertically, but for the 2nd and 3rd column they add up to 100% horizontally. I think just using the same logic across all columns makes most sense.*

To keep the logic consistent in the table, all percentages are calculated corresponding to the N of each column.

|  | 18-29 years old  N = 27 | 30-40 years old  N =23 | Total  N = 50 |
| --- | --- | --- | --- |
| Gender |  |  |  |
| Men | 16 (59%) | 8 (35%) | 24 |
| Women | 11 (41%) | 12 (52%) | 23 |
| Non-binary | 0 (0%) | 3 (13%) | 3 |
| Urban Residence | 23 (85%) | 18 (78%) | 41 |
| Employed | 16 (59%) | 17 (74%) | 33 |
| Post-Secondary Education | 27 (100%) | 23 (100%) | 50 |

*4. It’s not clear why there are two different formats for participant codes (e.g. p. 9 has “FG2 F” and “A109”.) Also not sure if using the codes adds anything over, say, using gender & age of the participant, but that’s just a though – just using a unified set of codes throughout is also fine.*

To keep participants’ codes consistent, we have unified our identification coding where all participants were identified by the focus group they were in and their gender. The following sentence was added in Method page 5 to clarify:

“Each participant was identified in the transcript by the focus group they participated in and the gender of the participant.”

*5. The name of the virologist with a podcast (p. 18) is misspelled. It should be “Drosten”. I’d also consider citing the podcast as it is mentioned as an exemplary form of outreach.*
The virologist’ name had been corrected (page 19) with reference added.

41. NDR. Das Coronavirus-Update von NDR Info [Internet]. Norddeutscher Rundfunk. 2020 [cited 2021 Jun 20]. Available from: /nachrichten/info/podcast4684.html
